# Rapid assessment of West Nile virus circulation in a German zoo based on honey-baited FTA cards in combination with box gravid traps

**DOI:** 10.1186/s13071-021-04951-8

**Published:** 2021-09-06

**Authors:** Noelle Fynmore, Renke Lühken, Heike Maisch, Tina Risch, Sabine Merz, Konstantin Kliemke, Ute Ziegler, Jonas Schmidt-Chanasit, Norbert Becker

**Affiliations:** 1 Institute of Dipterology (IfD), Georg-Peter-Süß-Str. 3, 67346 Speyer, Germany; 2 Faculty of Biosciences, Heidelberg University, Im Neuenheimer Feld 230, 69120 Heidelberg, Germany; 3 The Royal (Dick) School of Veterinary Studies, University of Edinburgh, Easter Bush Campus, Midlothian, EH25 9RG UK; 4 Department of Arbovirology, Bernhard-Nocht-Institute for Tropical Medicine, Bernhard-Nocht-Str. 74, 20359 Hamburg, Germany; 5 Faculty of Mathematics, Informatics and Natural Sciences, Universität Hamburg, Hamburg, Germany; 6 Thüringer Zoopark Erfurt, Am Zoopark 1, 99087 Erfurt, Germany; 7 Friedrich-Loeffler Institut, Institute of Novel and Emerging Infectious Diseases, Südufer 10, 17493 Greifswald-Insel Riems, Germany

**Keywords:** West Nile virus, Arbovirus, Surveillance, Culicidae, Saliva, Public health, Veterinary health

## Abstract

**Background:**

For over a decade, monitoring of West Nile virus (WNV) in Germany has consisted of a bird monitoring programme as well as a mosquito-based surveillance programme employing CO_2_-baited encephalitis vector surveillance (EVS) traps for mass trapping and screening of mosquitoes. In contrast to the EVS traps, the Reiter/Cummings type box gravid trap collects gravid female mosquitoes, which have already taken a blood meal, increasing the likelihood of being infected with pathogens. The traps can be equipped with a honey-baited Flinders Technology Associates^®^ (FTA) card to encourage sugar feeding by the trapped mosquitoes. FTA cards contain nucleic acid preserving substances, which prevent the degradation of viral RNA in the expectorated mosquito saliva and allows for testing the card for flavivirus RNA. This study aimed to assess the suitability of the method for WNV surveillance in Germany as an alternative to previous methods, which are expensive, time-consuming, and predominantly target host-seeking populations less likely to be infected with WNV.

**Methods:**

In the Thüringer Zoopark Erfurt, snowy owls (*Nyctea scandiaca*) and greater flamingos (*Phoenicopterus roseus*) died of WNV infections in July and August 2020. In response, five Reiter/Cummings type box gravid traps were positioned during the daytime on the 10th, 13th, and 16th of September in five different locations. The FTA cards and mosquitoes in the chamber were collected, kept in a cool chain, and further processed for virus detection using a modified generic flavivirus reverse transcription PCR.

**Results:**

A total of 15 trappings during September collected a total of 259 female mosquitoes, 97% of which were *Culex pipiens* sensu lato, as well as 14 honey-baited FTA cards. Eight mosquitoes tested PCR-positive for WNV. Four FTA cards tested PCR-positive for mosquito-borne flaviviruses, two of which were confirmed as WNV, and the remaining two confirmed as Usutu virus.

**Conclusion:**

The suitability of the FTA cards in preserving viral RNA in the field and rapid turnaround time from collection to result is combined with a simple, cost-effective, and highly specific trapping method to create an arbovirus surveillance system, which circumvents many of the difficulties of previous surveillance programmes that required the analysis of mosquitoes in the laboratory.

**Graphical Abstract:**

**Figure:**
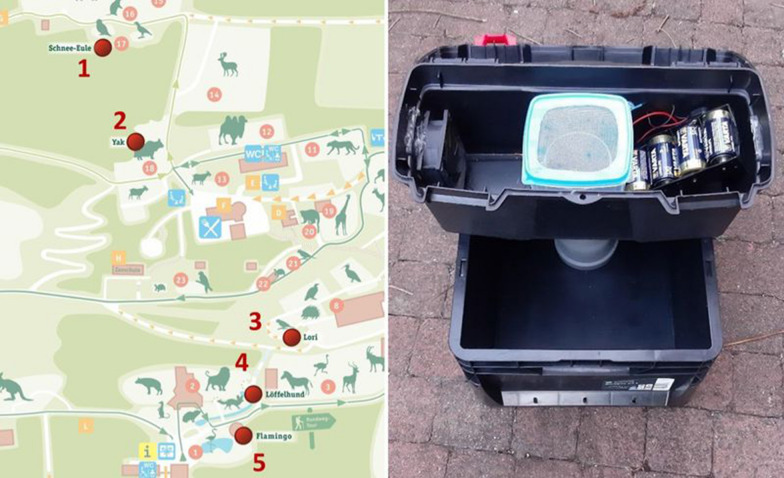

## Background

West Nile virus (WNV) is a member of the family *Flaviviridae* and belongs to the genus *Flavivirus* [1]. WNV circulates in an enzootic cycle with birds as amplifying host and mosquitoes as vectors [2]. The major vector species in Germany belong to the genus *Culex*, with *Culex pipiens* biotype *pipiens*, *Culex pipiens* biotype *molestus*, and *Culex torrentium* proven susceptible [3, 4].

Before 2018, there was no extensive autochthonous WNV circulation detected in Germany in any host populations, human or animal. Neutralising antibodies were predominantly detected in migratory birds [5–8]. WNV has the potential to become a considerable human health risk, especially for transfusion and organ transplantation safety, with cases of neuroinvasive disease resulting from infected transplanted organs approaching 75% in the USA [9]. In addition, PCR screening of blood donations from endemic WNV areas is recommended but causes additional costs.

Monitoring of birds for WNV has been conducted in Germany for over 10 years [5–7, 10], but the first confirmed cases were only detected during an outbreak in 2018 and included blackbirds (*Turdus merula*), northern goshawks (*Accipiter gentilis*), and great grey owls (*Strix nebulosa*), as well as horses [8]. In 2019 and 2020, the emergence of WNV was again observed in birds and horses, but for the first time several autochthonous human cases were also registered, including one fatal case in 2020 [11, 12]. As an alternative to dead-bird monitoring, mosquito surveillance could provide an opportunity to detect enzootic WNV circulation [13]. However, integrated arbovirus surveillance systems are time- and cost-intensive and not widely implemented in Europe [13]. In Italy, an integrated surveillance system for WNV detected WNV RNA in mosquitoes before the first cases in humans or animals were detected [14]. In addition, mosquito surveillance could be used to forecast spatial–temporal transmission risk [10].

In 2007, the first mosquito-based WNV surveillance programme was implemented by the German Mosquito Control Association and employed CO_2_-baited encephalitis vector surveillance (EVS) traps in major bird breeding, resting, and roosting habitats (hotspots) in the Upper Rhine Valley. In 2007 and 2008, more than 11,000 host-seeking adult female mosquitoes (13 species) were tested for WNV by the VecTest WNV antigen assay. All tests were negative for WNV [3]. Subsequently, a country-wide arbovirus surveillance network was established with a particular focus on WNV, which collects mosquito samples from a network of some 120 fixed position traps, and conducts surveillance studies on vertebrate species affected, such as birds and equines [1]. The mosquito traps employed include EVS traps, gravid and ovitraps, and BG-Sentinel CO_2_ traps and are set up in areas of ecological or epidemiologic importance [1]. Extremely large quantities of mosquitoes must be collected and maintained in a cool chain for transport, identification, and processing to preserve RNA [13]. The collection of large numbers of non-target species can result in additional handling and sorting being required [15].

In contrast to EVS style traps, gravid traps predominantly collect gravid female mosquitoes, which have likely already taken a blood meal, thereby increasing the likelihood that they are infected with pathogens [16, 17]. An illustration of how much more effective gravid traps can be in catching infected mosquitoes is seen in a trap comparison for WNV surveillance carried out in the USA, where gravid traps were found to have an infection rate 33 times higher than standard light traps with 2.29 versus 0.07 infected *Culex* mosquitoes per 1000 specimens [18].

Due to their ability to attract gravid females searching for an oviposition site, specifically, the CDC gravid trap or Reiter gravid trap has become popular for the collection of gravid female *Culex* mosquitoes in urban and suburban environments. However, the initial design resulted in a loss of 20% of the sample [17]. Modifications by Cummings [17] involved housing a collection chamber in a toolbox and mounting the fan and exhaust separately on the side of the toolbox so that mosquitoes were drawn into the chamber by the vacuum created but at no point passed through the fan.

In 2020, Thüringer Zoopark Erfurt housed 24 different bird species with 126 specimens. As part of the veterinary prophylaxis plan, dead wild birds found on the zoo grounds and dead zoo birds are investigated for WNV, Usutu virus (USUV), and avian influenza on a routine basis by the local state veterinary laboratories. In the course of the monitoring scheme, the first case of WNV in the zoo was detected in 2020. On the 12th of July, a female snowy owl (*Nyctea scandiaca*) died after several days of sickness. WNV was detected by RT-qPCR and confirmed by the national reference laboratory for WNV at the Friedrich-Loeffler Institute (FLI). In the last two weeks of August, further WNV-positive birds were detected. Another snowy owl as well as a juvenile greater flamingo (*Phoenicopterus roseus*) died after a period of symptomatic infection. Additionally, an adult female greater flamingo was found dead in the enclosure despite showing no signs of illness beforehand. The WNV genome was detected in different organs after the autopsy, and the findings were verified by the FLI. In addition, two keas (*Nestor notabilis*) and one rainbow lorikeet (*Trichoglossus moluccanus*) showed a loss in appetite, became lethargic, and lost weight at the end of August 2020. Blood samples were negative for the WNV genome, but all three birds showed specific neutralising antibodies against WNV and survived.

The active WNV-circulation built the ideal scenario to evaluate a new method to monitor mosquito-borne viruses in Central Europe. A technique developed by Hall-Mendelin et al. [19] has shown potential for significant improvements in both time and costs saved in mosquito-based disease surveillance programmes. The design takes advantage of how mosquitoes expectorate saliva when sugar feeding and offers trapped mosquitoes honey on a nucleic acid-preserving substrate, such as a Whatman^®^ Flinders Technology Associates^®^ (FTA) card [19]. Honey-baited FTA^®^ cards can easily be incorporated into the collection chamber of a Reiter/Cummings box gravid trap, and such gravid traps have already been successfully used with FTA cards in areas of low prevalence, such as in Switzerland [13]. This study aimed to assess the suitability of this method for WNV surveillance in Germany as an alternative to previous methods, which are expensive, time-consuming, and predominantly target a host-seeking subpopulation least likely to be infected with WNV.

## Methods

### Mosquito trapping with the Reiter-Cummings gravid trap, including FTA cards

**Fig. 1:**
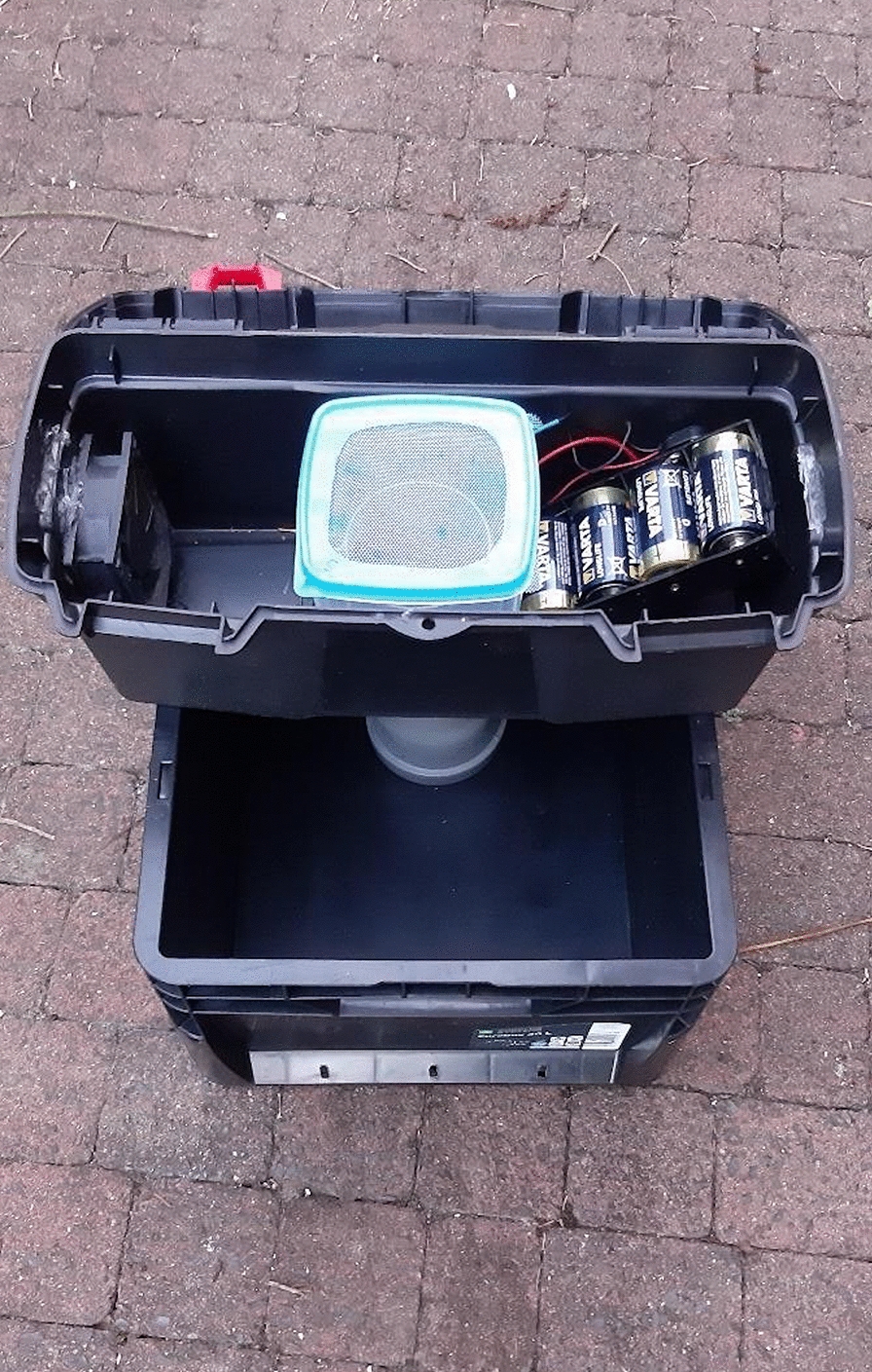
Modified Reiter/Cummings type box gravid traps with honey-baited FTA cards

Box gravid traps were constructed based on the design of the Reiter-Cummings gravid trap [17, 20]. Traps consisted of a lower tray in the form of a stackable black plastic box (Euronormbox 20 L, Surplus Systems GmbH, Bonn, Germany) (40 × 30 × 22 cm) and the main trap body consisting of a black plastic LUX Tools toolbox (Emil Lux GmbH & Co. KG, Wermelskirchen, Germany) (17 × 37 × 19 cm) (Fig. 1). Two holes with a diameter of 7.5 cm were drilled centrally into the bottom and left-hand side of the toolbox using a hole saw. The lower hole was used to insert a 15 cm long piece of polypropylene Marley HT Pipe DN 75 (Marley Deutschland GmbH, Wunstorf, Germany), 7.5 cm in diameter forming the intake pipe. Inside the toolbox, a small axial cooling fan (LogiLink DC Brushless Fan, Model Fan101, 2direct GmbH, Schalksmühle, Germany) was glued to the inside of the left hole. The fan was run with 4 D cell batteries, with the wiring and battery stowed within the toolbox casing, allowing a run time of 216 h.

A collection chamber for the mosquitoes was created out of a clear plastic food storage container. A large central panel from the lid was cut out and replaced with a 1.5 mm insect screen. A 7.5 cm diameter hole was cut into the bottom of the container to be mounted over the intake pipe. With the toolbox closed and the fan running, the air was drawn into the trap via the intake pipe, through the collection chamber, and blown out the side.

The main trap body was positioned over the lower tray and the lower tray filled with 5 L of liquid attractant, consisting of a simple hay infusion with 0.9 kg hay to every 114 L of water [21]. The intake pipe was positioned approximately 4 cm over the water surface.

Whatman™ FTA™ Classic cards (GE Healthcare Life Sciences, Buckinghamshire, UK) (FTA cards) were cut into quarters and prepared using a similar procedure to Wipf et al. [13]. The cards were affixed to the side of the trap in small 3 × 4 cm resealable sample bags. A 1.5 cm square hole was cut in the front of the bags to provide an access point to the surface of the FTA cards. Honey (Goldland Blütenhonig, W.L. Ahrens GmbH & Co. KG, Paderborn, Germany) was dyed with Brilliant Blue FCF (Blue 1) food colouring powder (Brillantblau FCF [C.I. 42090], Carl Roth GmbH & Co. KG, Karlsruhe, Germany) at a ratio of 1:100. A cotton pad (dm-drogerie markt GmbH & Co. KG, Karlsruhe, Germany) was placed behind the FTA card and soaked in a 1:10 ratio of blue-dyed honey and water to keep the FTA card moist during deployment. The FTA card was also soaked face down overnight on blue-dyed honey before being inserted into the resealable bag with the cotton pad for deployment.

To retrieve the sample from the trap, a mesh cloth was inserted into the intake pipe while the fan was still running to ensure no mosquitoes could escape. The main body of the trap was then opened to access the collection chamber. The batteries were disconnected to stop the fan, and the collection chamber was carefully lifted from the intake pipe, ensuring the insertion site was covered with mesh to prevent mosquitoes from escaping. The entire collection chamber with its FTA card was frozen to euthanise the mosquitoes before the FTA cards were removed traps and put into clean resealable bags. Mosquito samples were tipped into glass vials, and the cold chain was maintained for the mosquito samples and FTA cards until processing in the laboratory.

Thüringer Zoopark Erfurt comprises an area of 63 ha in the north of the city of Erfurt. It is situated on a hilly area and consists of a mixture of forests, meadows, and a few natural and artificial ponds. The WNV-positive tested snowy owls are situated on the northern plateau of the Zoopark with no pond in the direct vicinity but with a larger rainwater collection area nearby at the yak enclosure. The flamingo enclosure is situated at the southern border of the zoo at the base of the hill, and the enclosure contains a shallow wading pond for the birds. Keas and lorikeets are situated in the middle of the Zoopark hill. Although the keas do not have any standing water adjacent to their enclosure and only a shallow pool cleaned twice per week, there is a natural pond, also containing small fish or amphibians, right in front of the lorikeet aviary.

**Fig. 2:**
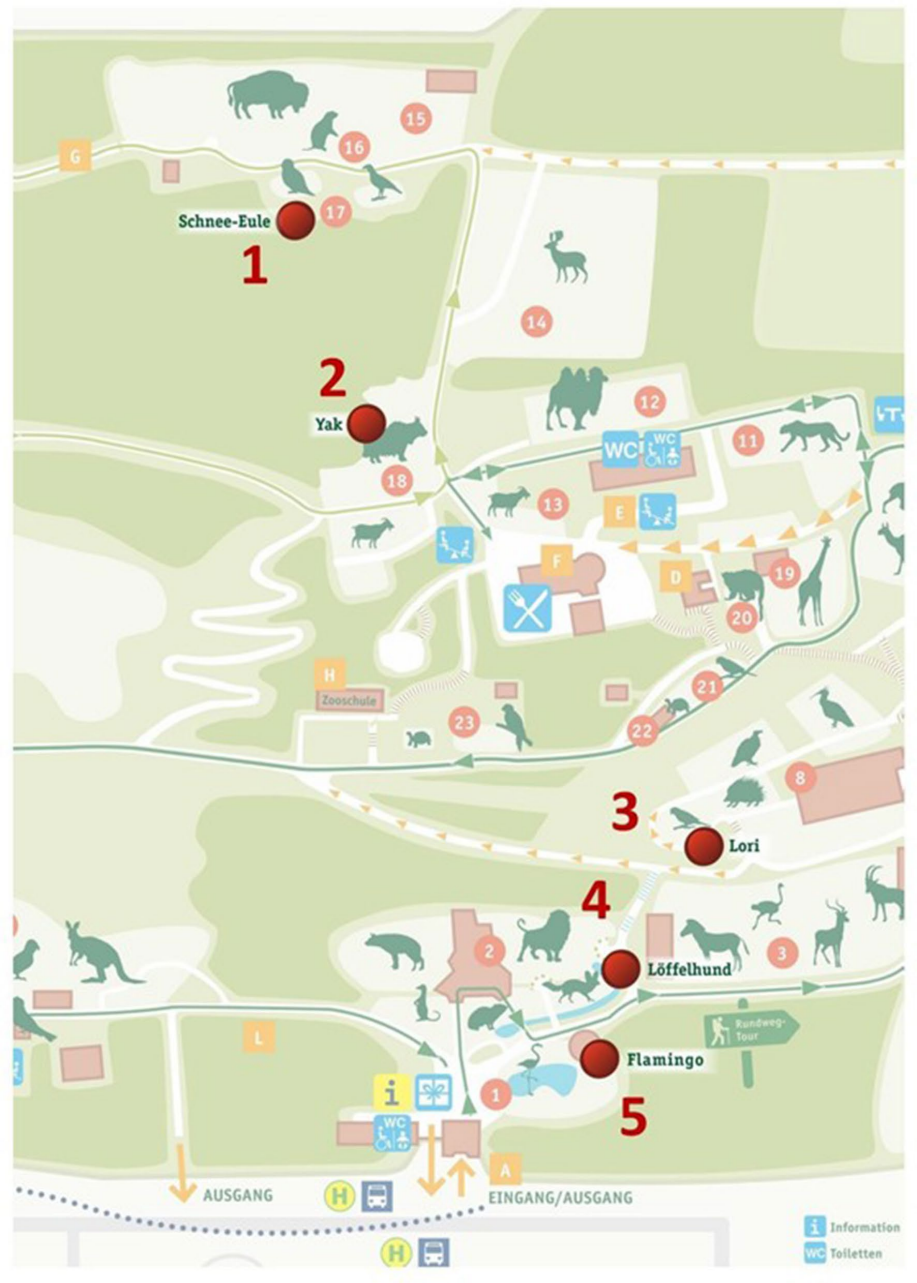
Locations of the Reiter-Cummings gravid traps in the Thuringian Zoo Park, Erfurt, Germany, close to the enclosures of 1: snowy owl; 2: yak; 3: lorikeet; 4: bat-eared fox; and 5: flamingo

Five traps were installed as follows: on the hill plateau directly next to the snowy owl aviary, in close vicinity to a pond on the plateau next to the yak enclosure, next to the lorikeet pond, next to the flamingo aviary, and next to the moat of the bat-eared foxes during the first cycle of catchments (Fig. 2). Traps were first set up on the 10th of September 2020 in the evening. The fan was visually inspected each time to ensure that it was operating correctly. Traps were set during daytime hours and left to operate continuously for 48 h in their position.

Three 48 h trapping sessions were carried out with FTA cards and mosquitoes collected on the 12th, 15th, and 18th of September from each of the five trap locations. In parallel, all potential breeding sites for *Cx. pipiens* s.l. were mapped by zoo staff. 32 breeding sites were identified with a surface area of between 1 and 190 m^2^. Twenty-four of these breeding sites, with a combined surface area of 1538 m^2^, were treated on the 11th and the 25th of September 2020 with VectoBac G (activity 200 ITUs/mg, Valent BioSciences, USA). In the remaining eight breeding sites, the water was changed weekly. For each breeding site, a pre-measured quantity of granules, determined by the surface area of the breeding sites, was packed in small bags for hand application at a rate of 5 g VectoBac G/m^2^. The gravid traps were then set for an additional two 48 h trapping rounds on the 27th of September, and the and 30th of September for the purposes of population monitoring.

### Mosquito identification

Mosquitoes were identified on a chill plate with a stereomicroscope (Motic, SMZ-171, Germany) using the morphological key by Becker et al. [22], and the sample returned to the freezer after identification. The mosquitoes and the FTA cards were shipped by express parcel overnight in a polystyrene box with cooling elements to the Bernhard Nocht Institute for Tropical Medicine in Hamburg for virus screening.

### RNA extraction and PCR protocols

Mosquito specimens were individually placed into 2 ml tubes, and about 10 pieces of 2.0 mm zirconia beads (BioSpec Products, Bartlesville, USA), as well as 0.5 ml of cell culture medium (high-glucose Dulbecco’s modified Eagle’s medium; Sigma-Aldrich, St. Louis, MO, USA), was added. The homogenisation was performed with a TissueLyser LT (Qiagen, Hilden, Germany) for 2 min at 50 oscillations/s.

FTA cards were individually placed into 2 ml tubes with 1 ml phosphate-buffered saline (PBS; PAN Biotech, Aidenbach, Germany). The tubes were kept on ice for 30 min and vortexed three times. We used 200 μl of the mosquito homogenates and PBS solution of the FTA cards for RNA extraction, which was performed with KingFisher™ Flex Magnetic Particle Processor using MagMAX™ CORE Nucleic Acid Purification Kit (both Thermo Fisher Scientific, Waltham, MA, USA). Samples were tested for flavivirus RNA using a modified generic flavivirus reverse transcription (RT)-PCR [10]. All amplicons were visualised on 2% agarose gels and PCR products sequenced with LGC Genomics (Berlin, Germany). Sequences were visualised and edited with Geneious version 9.1.7 (Biomatters, Auckland, New Zealand). The resulting sequences were submitted for virus species identification using the Basic Alignment Search Tool (BLAST) in the GenBank DNA sequence database (https://blast.ncbi.nlm.nih.gov/).

## Results

**Fig. 3:**
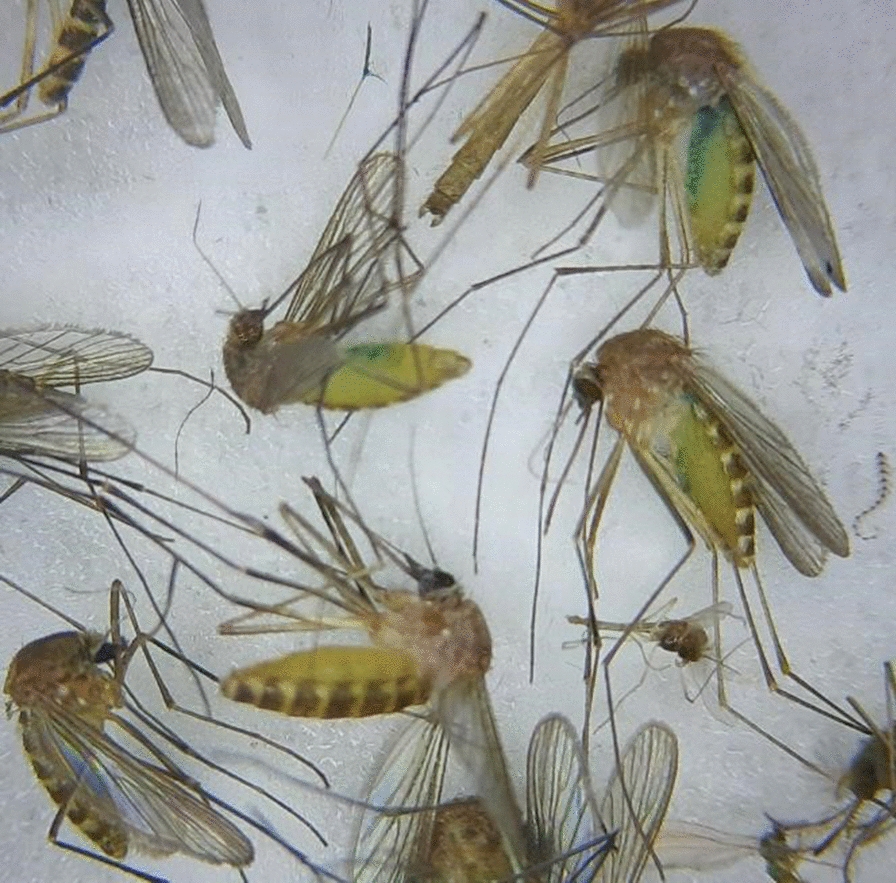
Example of mosquitoes showing blue colouring in the digestive tract as an indicator of feeding on the FTA cards

A total of 259 female mosquitoes were collected from the three trapping rounds. Of these, 142 females (55%) presented blue colouring in the digestive tract, which indicated feeding on the FTA cards (Fig. 3). Ninety-seven percent of specimens collected were *Cx. pipiens* s.l. (*n* = 251), with only extremely small numbers of *Culiseta annulata* (*n* = 6), *Anopheles plumbeus* (*n* = 1), and *Cx. modestus* (*n* = 1).

Of all mosquitoes collected, 11 mosquitoes (4% of all specimens) from four trapping positions tested positive for flavivirus RNA with the generic flavivirus RT-PCR (Table 1). In total, after the sequence analysis (nucleotide sequences can be obtained from the authors upon request), eight mosquitoes were positive for WNV lineage 2 (three specimens also fed from FTA cards), and three were positive for European USUV lineages (two specimens also fed from FTA cards).

**Table 1:** Overview of mosquitoes caught by trap site and numbers of which tested positive for WNV or USUV

Trap site	No. of mosquitoes collected	WNV-positive mosquitoes	USUV-positive mosquitoes
1	7	0	0
2	45	1	1^a^
3	5	1	0
4	131	5^a^	2^a^
5	71	1	0
Total	259	8	3

^a^These locations had a correspondingly positive FTA card

**Fig. 4:**
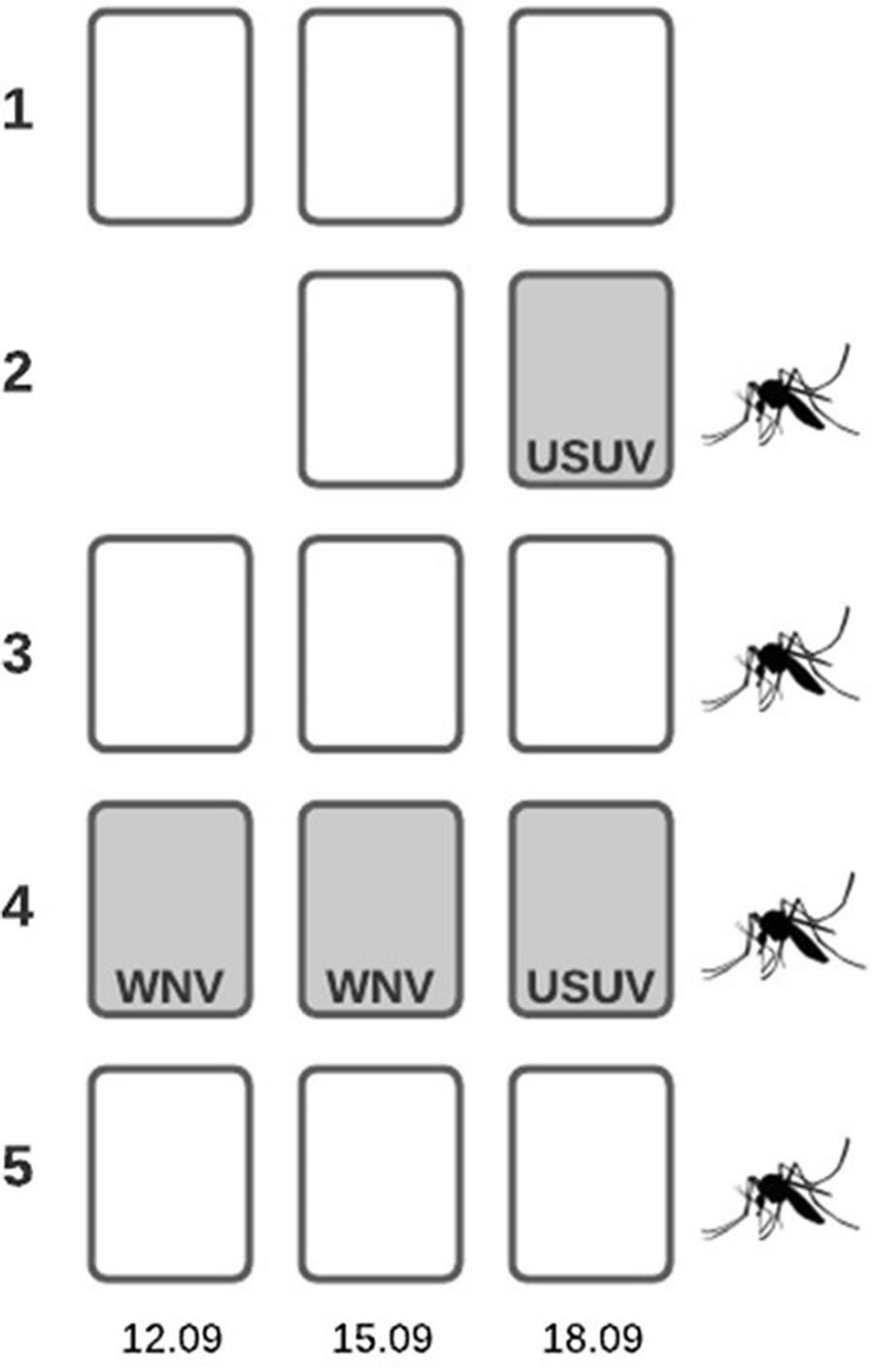
FTA card results by sampling location. Mosquitoes represent locations where the mosquito sample contained mosquitoes positive for flaviviruses

Fourteen FTA cards were collected, and 29% (*n* = 4) were positive for flavivirus RNA (Fig. 4). One card was unable to be retrieved for processing. Positive cards were collected from two trapping locations, which also had WNV- and USUV-positive mosquitoes. Site 2 near the yak enclosure had an FTA card that tested positive for USUV. Of the two positive mosquitoes collected from this location, one unfed (non-blue-coloured) mosquito specimen tested positive for USUV, and one fed, blue-coloured mosquito specimen tested positive for WNV. The second location near the bat-eared fox enclosure (site 4) was positive for USUV (one FTA card) and WNV (two FTA cards). Two fed mosquitoes from this trap were positive for USUV, and another five unfed mosquitoes were positive for WNV.

The remaining two flavivirus-positive locations had only a single fed mosquito, each testing positive for WNV in the trapping period. Neither of these traps had a correspondingly positive FTA card.

The two additional rounds of trapping in late September resulted in the collection of a total of 10 mosquitoes. These mosquitoes were not included in the main analysis.

## Discussion

With circulation at levels high enough to result in multiple autochthonous transmissions, this is the first time that FTA cards have been successfully used in Germany to detect WNV RNA. In this study, 3% of the mosquitoes and 14.3% of the FTA cards were positive for WNV RNA. In contrast, in 2019, out of 306 pools made up of 2241 mosquitoes, only 2.3% or seven pools tested positive [23]. The sampling effort required to ensure detection using the gravid trap method is therefore considerably reduced. Because individual mosquitoes were tested, the prevalence rate is likely higher than what could be estimated from the study mentioned above. Additionally, had the mosquitoes in this study been pooled, the percentage of positive pools would have approached 100%, highlighting the difficulty of interpreting pooled results with associated reduced data granularity.

Temperatures in 2018 and 2019 were unusually warm and dry, providing conditions suitable for the first autochthonous transmissions of WNV [8] with a reduced extrinsic incubation period as a driving factor [12]. As temperatures continue to increase, WNV epidemics in Germany will likely continue to occur and increase in severity. A mosquito-based surveillance system would enable detection of circulating virus before it results in human cases [1]. It is important that such a system is sensitive but allows for rapid processing time and subsequently implementation of control measures to minimise future risks to human and animal health, e.g., to inform blood banks to screen donations or conduct vector control measurements.

Potential benefits to the system as used here are twofold. Due to their efficiency in collecting gravid *Cx. pipiens* s.l., gravid box traps offer a suitable method to establish and monitor incidence rates of WNV. Such baseline data is an invaluable tool that can be used to set thresholds and conditions under which control programmes should be undertaken [15]. Additionally, there is potential for a rapid, baited FTA card system without analysing the trapped mosquitoes to be used similarly to sentinel animals [15, 24]. In a field trial of passive traps with honey-baited FTA cards in northern Australia, WNV was isolated even in the absence of seroconversion from sentinel chickens, suggesting that the method may be more sensitive than those using sentinel animals [24].

It is difficult, if not impossible, to ascertain the sensitivity of this method without a reliable control. Both Flies et al. [25] and Wipf et al. [13] put forward testing the captured mosquitoes for the virus as a form of control or to determine the vector involved where an FTA card is positive. However, Hall-Mendelin et al. [19] periodically found positive mosquitoes in traps without a positive FTA card, and this study also shows that it cannot be assumed that a negative FTA card will mean a negative pool of mosquitoes, nor that a negative pool of mosquitoes will correspond to a negative FTA card. Moreover, detection of an infected mosquito does not mean that the mosquito was infectious at the point when it was euthanised. This distinction is an important one when considering the aims of an early warning surveillance programme, which requires detecting infectious mosquitoes that can contribute to the transmission cycle.

Because expectorated RNA is inactivated and preserved for several days on the FTA cards, it provides the potential to leave traps running for a longer period in the field [19]. It has been hypothesised that an extended period could serve to increase the sensitivity of the FTA card method by providing time for completion of the extrinsic incubation period, allowing time for the viral load to increase in the vector, and increasing the amount of time mosquitoes have access to the cards for repeated probing and the amount of potentially infected expectorate on the cards [25]. Additionally, because only the FTA cards require analysis, damage to the mosquito sample through heat, mould, or the presence of ants or spiders does not affect the viability of the trap as a virus detection tool.

Laboratory experiments by Hall-Mendelin et al. [19] showed that viruses could be detected on FTA cards even when no blue colouring could be seen in the mosquito. In subsequent field trials by the same authors, the virus was detected on FTA cards several times, even in the absence of a positive mosquito sample. This seems to have occurred in our study, where one FTA card was positive for USUV despite no evidence of blue colouring in the USUV-infected mosquito from the same trap. Because *Cx. pipiens* s.l. has been shown to transmit the WNV virus through probing alone [26], it is feasible that this could occur in the future for WNV detections on FTA cards. Therefore, adding blue colouring to the honey may be a redundant step going forwards.

As the infection rate in mosquitoes increases through the summer season, the likelihood of detecting an infected mosquito will also increase [3]. Because the sample size is tied to the likelihood of detection, intensive surveillance from sentinel sites would be more effective for early warning disease surveillance than broad-scale sampling with inadequate sample sizes [27]. It would then be beneficial to place traps in areas that had high virus circulation in the previous season [28].

Surveillance for WNV needs to be sensitive and allow for early detection, and there remains a need for an early detection system for Europe [29]. Despite the relatively low number of mosquitoes collected overall in this study, a surprisingly high percentage of the specimens collected were positive for WNV. The frequency with which positive mosquitoes and positive FTA cards were found together despite low numbers of mosquitoes found overall suggests the method is relatively sensitive.

The FTA card system tested here is a straightforward surveillance method, which allows rapid screening. Therefore, it is ideal as an early warning system in areas with high infection pressure and can make a scaled-up version of a standard mosquito surveillance programme feasible [19]. Additionally, despite the low number of traps, extremely few mosquitoes were caught in the weeks following the two rounds of treatment of all standing water. It is the opinion of the authors that where there is active disease transmission, a fortnightly treatment with microbial larvicides based on *Bacillus thuringiensis israelensis* (Bti) should be undertaken to heavily reduce the local *Cx. pipiens* s.l. vector population.

## Conclusions

Combined with the high catch rate of gravid *Culex* mosquitoes, as well as the low cost and ease with which they can be constructed, box gravid traps constitute an effective trap design for the surveillance of flaviviruses, including WNV. The use of FTA cards within these traps resulted in multiple successful detections of viral RNA. The ease with which the FTA cards can be incorporated into a box gravid trap and the speed with which they can be processed lends the method well to a vector-based arbovirus surveillance system. In future, it offers the potential to bypass the mass collection and identification of mosquitoes entirely, especially where the sensitivity of the method can be proven.

## Data Availability

All data generated or analysed during this study are included in this published article.

## Abbreviations

EVS: Encephalitis virus surveillance
FTA: Flinders Technology Associates
RNA: Ribonucleic acid
RT-PCR: Reverse transcription polymerase chain reaction
USUV: Usutu virus
WNV: West Nile virus
